# Increased isolevuglandin-modified proteins in glaucomatous astrocytes

**Published:** 2009-06-01

**Authors:** Bharathi Govindarajan, Anna Junk, Mabel Algeciras, Robert G. Salomon, Sanjoy K. Bhattacharya

**Affiliations:** 1 Department of Chemistry, Case Western Reserve University, Cleveland, OH; 2 Bascom Palmer Eye Institute, University of Miami, Miami, FL

## Abstract

**Purpose:**

Lipid oxidation has been proposed to be a factor in the pathophysiology of glaucoma. We investigated whether elevated levels of isolevuglandin (iso[4]LGE_2_) protein adducts are associated with astrocytes derived from the glaucomatous optic nerve head. In addition, we examined whether the iso[4]LGE_2_ protein adducts are altered following exposure of astrocytes to elevated pressure.

**Methods:**

Astrocytes were isolated from rat brain cortex and human optic nerve and were subjected to pressure treatments, western blot analyses, liquid chromatography tandem mass spectrometry, and immunocytochemistry.

**Results:**

Elevated levels of isolevuglandin (iso[4]LGE_2_) protein adducts were associated with astrocytes derived from the glaucomatous (n=10) optic nerve head when compared to those from controls (n=6). Astrocytes subjected to in vitro pressure treatment resulted in increased levels of iso[4]LGE_2_ protein adducts. Pressure exposure and the recovery period affect iso[4]LGE_2_ protein modification, and pyridoxamine was effective in decreasing the appearance of iso[4]LGE_2_ protein adduct immunoreactivity when applied immediately after pressure treatment.

**Conclusions:**

These results suggest that the elevated iso[4]LGE_2_ protein adduct immunoreactivity observed in glaucomatous astrocytes may be due to chronic and/or prolonged exposure to pressure, and pyridoxamine may have prophylactic utility against such oxidative protein modification.

## Introduction

Primary open-angle glaucoma (POAG), a leading cause of blindness worldwide [1,2], is a progressive and irreversible disorder that is often accompanied by increased intraocular pressure (IOP) and characterized by optic nerve damage. Elevated IOP leads to damage of the axons of retinal ganglion cells and is responsible for their death. The initial site of optic nerve damage is believed to be the lamina cribrosa [3,4]. Changes in the damaged optic nerve head (ONH) involve several cell types including neurons and surrounding glial cells. Glial cells in the central nervous system (CNS) are divided into three major types: astrocytes, microglia, and oligodendrocytes [5]. The astrocytes, oligodendrocytes, and microglias protect the neurons from damage and have other supportive functions. Following injury to the CNS, the glial cells undergo activation and initiate repair processes.

Previous studies recorded changes in morphology as well as modulation of several molecules in response to an increase in hydrostatic pressure. For example, increased adenylyl cyclase activity [6], nitric oxide synthase 2 [7], elastin [8], and Neural cell adhesion molecule (NCAM) [9] were found to be upregulated in astrocytes from the optic nerve head and in parallel, in vitro in response to hydrostatic pressure.

Intraocular pressure is known to affect different cell types in the optic nerve head [10]. The median normal intraocular pressure in the eye is about 16.5 mmHg [11]. The average intraocular pressure in POAG patients is amazingly only about 23–24 mmHg with around 30% having a pressure that is in the statistically normal range (21 mmHg or less) [12-14]. It is not unusual to have an untreated pressure near 30 or 40 mmHg often in relatively young patients of pigment dispersion, pseudoexfoliation, or glaucoma that results from other causes. Nevertheless, in some cases of glaucoma, the arterial supply to the ciliary body is obstructed when the pressure reaches a level near 60 or 70 mmHg. At this pressure, the eye cannot make any aqueous humor, and the pressure ceases to rise to a higher level. In a few cases of people with high blood pressure, the intraocular pressure may reach 80 mmHg if the arterial pressure is high enough to keep the ciliary body nourished, but the maximum that can be achieved is around 60–70 mmHg (personal communication, Dr. Douglas Anderson, Bascom Palmer Eye Institute, Miami, FL) [12,13]. This contrasts with brain injury in which very high pressure is occasionally experienced by cells including astrocytes [15]. Previously, in studies that model brain injury, astrocytes were subjected to very high pressures, often more than five times that of atmospheric pressure for a short duration [16].

Oxidative stress has been implicated as an important factor influencing neurodegenerative diseases [17,18]. The eye and the brain are known to consume large amounts of oxygen that often exceed their antioxidant defense systems, resulting in the generation of free radicals and causing elevation in levels of oxidative stress. Reactive oxygen species (ROS) produced from the respiratory chain in the mitochondria are largely responsible for oxidative stress in the eye and brain [19,20].

**Figure 1:**
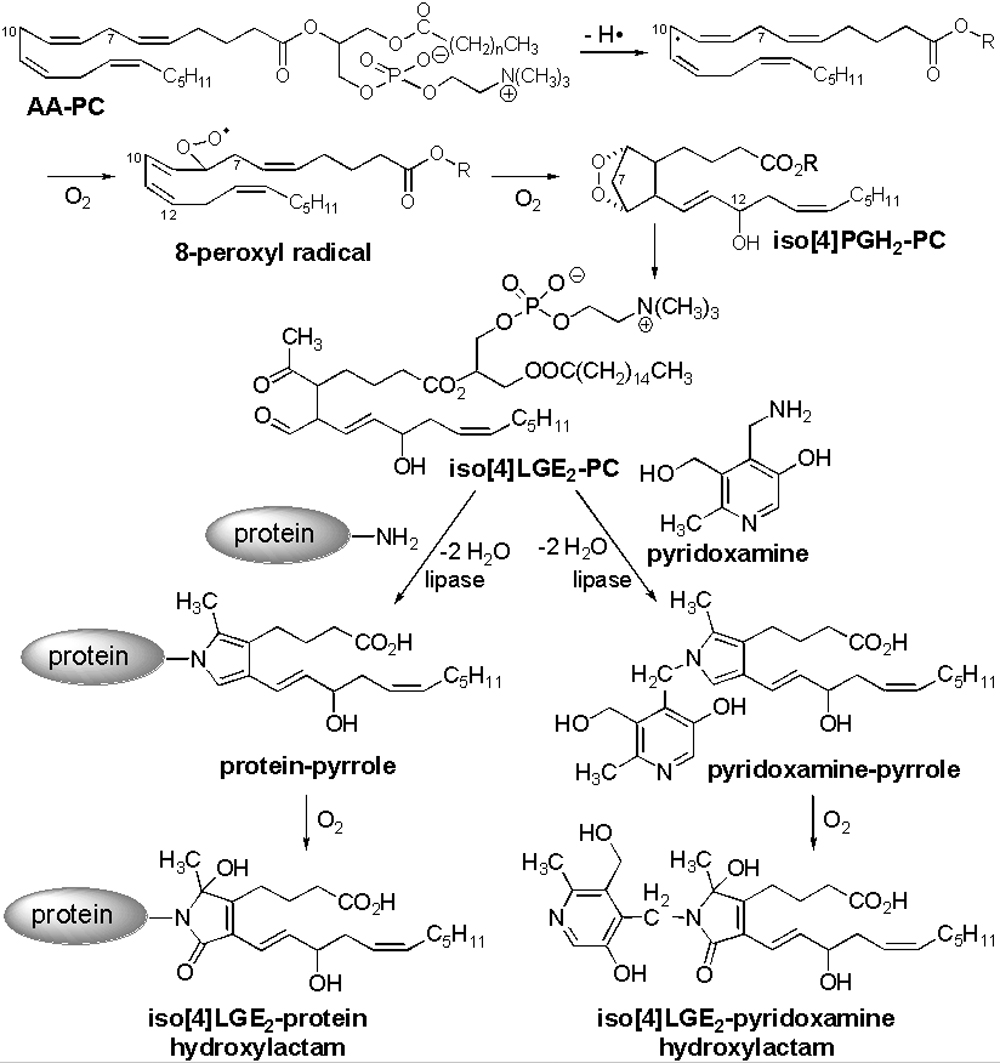
Schematic representation of isolevuglandins (isoLGs) generated by free radical-induced oxidation of arachidonates. Arachidonates such as AA-PC are efficiently trapped by pyridoxamine, preventing their adduction to proteins.

The elevated levels of oxidative stress in the eye and brain also make them likely targets for lipid peroxidation due to the presence of very high levels of lipids that incorporate arachidonic and docosahexaenoic acids [21-23]. Arachidonic acid was detected in the bovine optic nerve [24,25]. Isolevuglandins are produced by free radical oxidation of arachidonic acid (Figure 1) by the isoprostane pathway [26]. They are highly reactive γ-ketoaldehydes capable of adduction to lysine residues of proteins to form Schiff’s base adducts. Isolevuglandins have been implicated in neurologic disorders such as Alzheimer disease [27], multiple sclerosis [28], amyotrophic lateral sclerosis [29], and in glaucoma [30,31]. Previously, we demonstrated protein modification in glaucomatous ocular tissues by lipid peroxidation products including specific modification with an isolevuglandin, iso[4]LGE_2_ [30,31].

Astrocytes provide metabolic factors to the neurons and also help in the clearance of neuronal excretory products [32]. In POAG, astrocytes in the ONH are often exposed to elevated pressure due to elevation of IOP. Similarly, in many traumatic brain injuries, astrocytes are exposed to abnormally elevated pressure as well [16]. Pressure gradients have been proposed to play a major role in neuronal injury [33]. Following injury to the CNS, quiescent astrocytes become activated [34] and secrete growth factors and cell adhesion molecules such as platelet derived growth factor, several different proteoglycans, glial fibrillary acidic protein (GFAP), and vimentin [35,36]. Reactive astrocytes have been implicated in several neurodegenerative disorders such as Alzheimer and Parkinson [37,38] and in glaucomatous optic neuropathy [34]. Astrocytes migrate to the site of the damaged optic nerve in glaucomatous neuropathy and produce neurotoxic factors such as nitric oxide and tumor necrosis factor alpha [39,40]. Astrocytes but not neurons possess the capacity to synthesize arachidonic and docosahexaenoic acids [41].

The specific occurrence of iso[4]LGE_2_-modified proteins in astrocytes derived from glaucomatous eyes or CNS tissue exposed to over-pressure have not been investigated. Our initial analyses did not show a discernable difference in iso[4]LGE_2_ protein adduct immunoreactivity between control and glaucomatous optic nerve tissues. This is in contrast to the trabecular meshwork (TM) where a significant difference in iso[4]LGE_2_ protein adduct immunoreactivity was found between control and glaucomatous tissues [30,31].

We now present evidence that lipid oxidation products (iso[4]LGE_2_-modified proteins) are present in astrocytes derived from glaucomatous ONH. In addition, astrocytes subjected to pressure treatment develop iso[4]LGE_2_ modifications in vitro that may provide insight into the in vivo modification events. We further demonstrate that pyridoxamine, a pharmacological agent and known inhibitor of iso[4]LGE_2_ formation [42], can protect astrocyte proteins against modification by iso[4]LGE_2_, which is generated endogenously upon pressure treatment.

## Methods

### Isolation of astrocytes and pressure treatment

Astrocytes were obtained either from the human optic nerve or from rat brain cortex tissue. For human ONH astrocytes, cadaver donors were procured. All donor eyes were from Caucasian individuals (except one Asian 66-year-old female glaucomatous donor) from either gender who were subjected to enucleation within 12 h of death following the Tenets of Helsinki using previously described methods [30,43]. The donor eyes were received from donors of two age groups, 7−10 years old for one group and 54−84 years old for the other group. Donor eyes for these experiments were procured from the National Disease Research Interchange (Philadelphia, PA), Florida Lions Eye Bank, Miami, FL, and the Cleveland Eye Bank (Cleveland, OH). Astrocytes were also obtained from the brain cortex of Sprague Dawley rats (Harlan, Indianapolis, IN), from postnatal day 3 (P3) pups and enriched at 99% purity following previously published protocols [44]. Cells from rats were procured following the approval of the Institutional Animal Care and Use Committee, and the procurement adhered to the tenets of the ARVO statement. Briefly, the cerebrum was removed under a dissection microscope and placed in Hank's Buffered Salt Solution (HBSS) buffer containing 1% penicillin/streptomycin/amphoterecin (Invitrogen Inc., Carlsbad, CA). The mixed glial cell suspensions were grown on 75 cm plates coated with 5 µg/ml poly-l-lysine (Sigma Chemical Co., St. Louis, MO) in Dulbecco’s modified Eagle’s medium (DMEM) containing 10% fetal bovine serum (FBS) and penicillin and streptomycin [45]. From these mixed cell suspensions, C5-positive cells were obtained using immunopanning [46]. Cells were subsequently cultured in Ham’s F-10 medium with 8% FBS, subjected to passage with a 0.25% trypsin solution, and subsequently plated in a serum-free astrocyte growth medium (AGM) [47]. After 24 h in culture, the medium was changed to AGM containing 5% FBS. The majority of the cells that attached in the serum-free medium were astrocytes. Sequential panning was adopted to further purify astrocytes. Briefly, the cell suspension of primary cells was panned first on a Petri dish coated with C5 monoclonal antibody to select for cells of astrocyte lineage using multiple rounds of C5 based cell enrichment through immunopanning [46].

Change to: Isolated astrocytes were plated on 15 mm culture plates and subjected to pressure in a sealed pressure chamber [44,48] for pressure and duration as indicated in each individual experiment. After pressure treatment, the cells were allowed to settle down in an incubator at 37 °C with 5% CO2/95% air for a period of 18–24 h unless stated otherwise. The plates were subjected to trypsin treatment for 10 min and centrifuged at 10,000x g for 10 min. The cells were collected and resuspended in 10 µl of 1X phosphate buffered saline (PBS) and added to 25 µl of lysis buffer (25 mM Tris pH 7.5, 100 mM sodium chloride [NaCl], 5 mM dithiothreitol [DTT], 1 µl of 50 mM disodium hydrogen phosphate [Na_2_HPO_4_], 1 µl of 1 mM diethylenetriaminepentaacetic acid [DTPA], 1 µl of 100 µM butylated hydroxy toluene [BHT] and 10% sodium dodecyl sulfate [SDS]). The cells were then subjected to vortexing for 1 min, boiled, and used immediately or stored at −20 °C for future use.

### Western analyses

Protein was quantified using the biochinchonic acid (BCA) protein assay (Pierce Biotechnology Inc., Rockford, IL). Western blot analysis was performed with 25 µg of protein on 4%–20% Tris-glycine gels (Invitrogen Inc.). After fractionation, the proteins were electroblotted onto a polyvinylidene fluoride (PVDF) membrane (Millipore, Billerica, MA) using standard procedures and probed with the rabbit polyclonal iso[4]LGE_2_ protein adduct antibody. The characterization of iso[4]LGE_2_ protein adduct antibody has been described in detail in a previous report [49].

### Enzyme-linked immunosorbent assay

About 5 µg of purified bovine serum albumin (BSA) or 10 µg of astrocyte lysate was centrifuged at 10,000x g for 10 min, and the resulting clear solution was transferred, placed in individual wells in a plate (Costar 9018 plate; eBioscience, Inc., San Diego, CA), and incubated for 20 min at room temperature. The supernatant was discarded, and the plate was washed with PBS. The plates were blocked with 1% ovalbumin for 1 h, washed with PBS, and incubated for 1 h with rabbit polyclonal antibody against iso[4]LGE_2_ protein adduct. After subsequent washes with PBS, plates were incubated with the secondary antibody coupled with alkaline phosphatase for 1 h, washed with PBS, and incubated with phosphatase substrate (100 µl/well) in diethanolamine buffer, pH 7.5. The absorbance was then measured at 405 nm on a plate reader. The results of the enzyme-linked immunosorbent assay (ELISA) analysis are expressed as mean±standard error of the mean. Statistical analysis was performed for each sample at every experimental point shown, was compared to 0.0 using the two tailed one-sample t-test, and was found significantly different from 0.0 at each time point by the one-sample t-test (*p<0.05). The asterisk indicates that the p value is less than 0.05.

### Immunoprecipitation

About 3,000 astrocytes were placed in 100 µl of lysis buffer (1X PBS with 0.1% Genapol). The cells were lysed by subjecting them to two cycles of freeze–thaw at −80 °C and 37 °C, respectively. The protein concentration of the lysate was determined using the BCA assay method described above. About 50 μl of protein A beads were coupled with 10 μg of the iso[4]LGE_2_ protein adduct antibody using 20 mg of dimethyl pimelimidate following suitable modifications of established protocols [50]. The antibody coupled beads were prewashed with 500 µl of lysis buffer, subsequently incubated with 50 µl of cell lysate (~100 µg protein) in a total volume of 250 µl, and then allowed to incubate overnight at 4 °C. After washing the beads three times with 500 µl lysis buffer, the products were eluted from the beads with 30 µl of 100 mM glycine, pH 3.0. The eluents were added to 5 µl of Laemmli buffer, boiled for 1 min, and then separated over a 4%–20% gradient gel. The gels were fixed with a 4:5:1 ratio of methanol:water:acetic acid, washed with distilled water, and subsequently stained with Gel Code Blue solution (Pierce Biotechnology Inc., Rockford, IL). The control as well as the 25 mmHg and 100 mmHg treated cells were subjected to anti-iso[4]LGE_2_ protein adduct and SDS–PAGE separation. The proteins bands were subsequently excised, in-gel trypsin digested, and subjected to tandem mass spectrometry.

### Treatment with pyridoxamine

In an effort to block protein modification by the lipid peroxidation product iso[4]LGE_2_, pressure-treated astrocytes were subjected to incubation in the presence of pyridoxamine, an inhibitor of the isolevuglandin protein adduct and of advanced glycation end product formation [42]. The experiment was performed using a range of pyridoxamine concentrations (5–50 mM) to observe the changes in isolevuglandin modification of proteins. Control astrocytes were those not subjected to either pressure or treatment with pyridoxamine. About 3,000 astrocytes were plated on 15 mm plates and grown overnight in DMEM at 37 °C. Cells grown overnight were treated with pyridoxamine (Sigma Chemical Co.) in 4 ml DMEM so that the final pyridoxamine concentration reached 5 mM, 15 mM, or 50 mM. These cells were incubated with pyridoxamine before pressure treatment and after being subjected to 100 mmHg pressure for 3 h. All pressure-treated cells were incubated at atmospheric pressure for 16 h at 37 °C for post pressure recovery. The cells were subjected to lysis by placing them into 100 µl of lysis buffer (1X PBS and 0.1% Genapol) and subjecting them to two freeze–thaw cycles at −80 °C and 37 °C, respectively. After centrifugation at 10,000x g for 10 min, the cell lysates were subjected to SDS–PAGE fractionation and western blot analysis with rabbit polyclonal anti-iso[4]LGE_2_ protein adduct.

### Cytochemical analysis and microscopic imaging

The isolated astrocytes were allowed to settle overnight at 37 °C at atmospheric pressure on coverslips placed in a 15 mm culture dish containing DMEM. For pressure treatment, the plates were placed in the pressure chamber at a specific pressure for a given duration specified for individual experiments. Following incubation, they were placed for recovery in the incubator at 37 °C and at atmospheric pressure. After recovery, cells were fixed for 30 min in 100 μl of 4% paraformaldehyde in PBS (pH 7.5) and were washed using 100 μl of 1X PBS (pH 7.5), which contained 80 μl of 0.2% Triton X-100 for 30 min. The cells were incubated with mouse monoclonal antibody against GFAP (1:200 dilution) and rabbit polyclonal anti-iso[4]LGE_2_ (1:200 dilution) [49] overnight at 4 °C. The secondary antibodies, anti-mouse and anti-rabbit, were coupled with Alexa 488 and Alexa 594, respectively. Following incubation with secondary antibodies, the cells were stained with 4',6-diamidino-2-phenylindole (DAPI) for 45 min. Astrocytes were also stained with phalloidin to observe changes in the actin filaments. For this purpose, the pressure-treated and the untreated control cells were fixed and permeabilized using 0.1% Triton X-100 for 1 min. The cells were then stained with rhodamine-phalloidin (1:100 dilution in 1X PBS) for 5 min. Following staining, the coverslips were inverted and placed on slides, sealed with Vectashield (Vector Labs, Burlingame, CA) and subjected to fluorescence microscopy on a Nikon microscope, model EFD-3 (Diagnostic Instruments Inc., Sterling Heights, MI).

## Results

### Isolated astrocytes from glaucomatous cadaver donors showed elevated immunoreactivity for iso[4]LGE_2_ protein adduct

**Figure 2:**
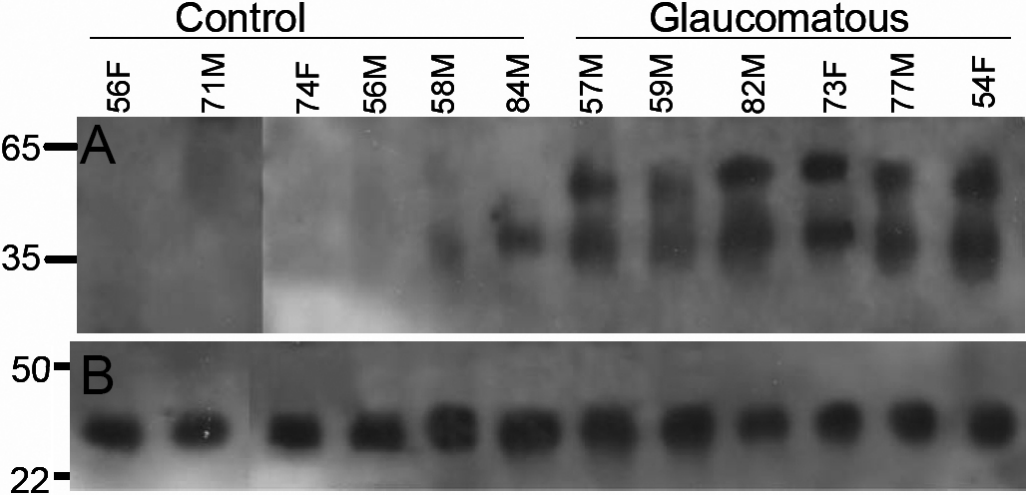
Increased iso[4]LGE_2_ modification of proteins in astrocytes from glaucomatous optic nerve head. A representative western blot analysis is shown of total protein lysates (25 µg) from astrocytes derived from the human optic nerve head. Age and gender of the donors (all Caucasian individuals) have been depicted. **A** was probed with rabbit polyclonal antibodies to iso[4]LGE_2_–protein adduct, and **B **was re-probed with mouse monoclonal antibody to GAPDH.

Isolated astrocytes derived from the optic nerve head of patients with POAG showed an increase in iso[4]LGE_2_ protein adduct immunoreactivity (Figure 2A) Two significant bands with molecular weights (MWs) of ~35 and ~50 kDa were observed to have modifications in the glaucomatous optic nerve head astrocytes. A total of 10 glaucomatous donors and six normal donors were probed for this study. All glaucomatous donors had elevated iso[4]LGE_2_ protein adduct immunoreactivity as shown in the representative blot for six Caucasian donors of either gender (Figure 2A). All donors were Caucasian except one glaucomatous Asian donor. This is similar to the trabecular meshwork where a significant increase in iso[4]LGE_2_ protein adduct immunoreactivity was found in tissues from glaucomatous eyes [30,31].

### Increased pressure induces iso[4]LGE_2_ modifications in astrocytes

**Figure 3:**
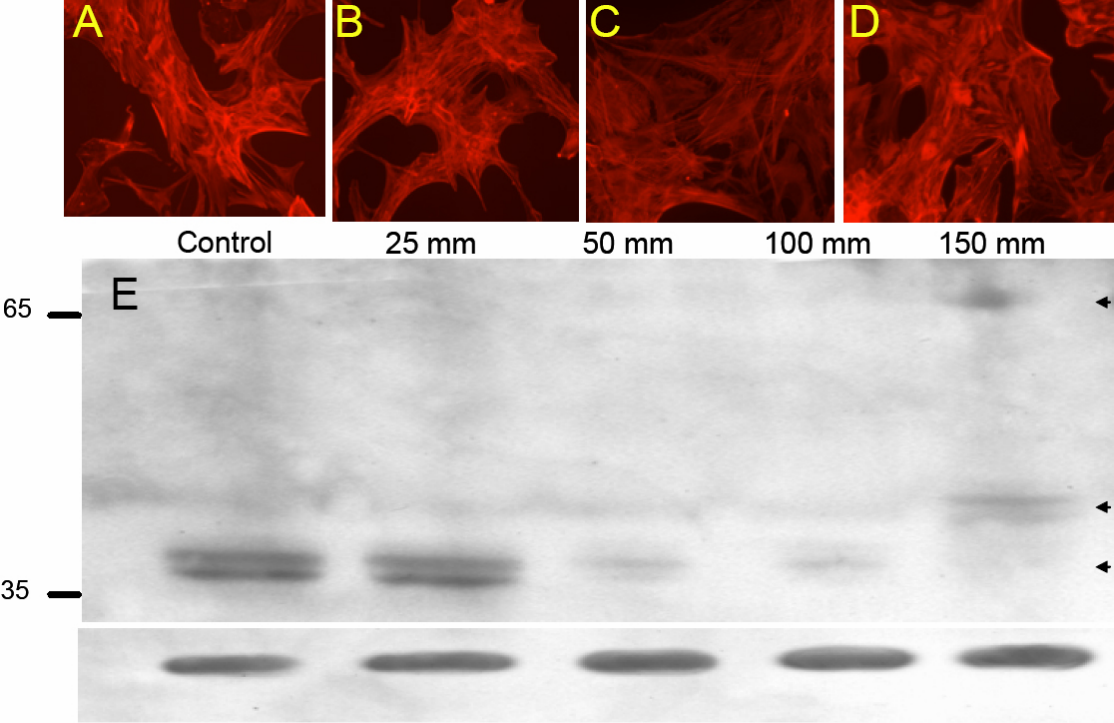
Analyses of astrocytes subjected to pressure. Representative fluorescence microscopic images of astrocytes are shown. About 3,000 isolated rat brain cortex astrocytes were subjected to pressures. Astrocytes were stained with rhodamine-phalloidin to determine the integrity of the actin cytoskeleton after 3 h of pressure treatment at the indicated pressure and a following 16 h recovery period: control cells (**A**) without pressure treatment, cells subjected to 25 mmHg (**B**), cells subjected to 100 mmHg  (**C**), and cells subjected to 150 mmHg (**D**). All these cells were subjected to fluorescence microscopy with iso[4]LGE_2_–protein adduct antibody after pressure treatment and control cells that did not go through any pressure treatment. **E**: Western blot analysis for iso[4]LGE_2 _modification is displayed. Astrocytes were subjected to pressures (25–150 mmHg) for a period of 3 h at 37 °C. Bottom panel was probed with anti-GAPDH antibody.

**Figure 4:**
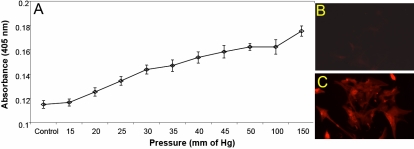
Analyses of astrocytes subjected to pressure for iso[4]LGE_2_–protein adduct formation. **A**: ELISA analysis was performed with antibodies to iso[4]LGE_2_–protein adduct and 10 µg of the control and pressure-treated astrocyte lysate (described in greater detail in Methods). Following pressure treatment, the culture medium was immediately replaced with fresh medium, and the cells were incubated at 37 °C for 16 h at atmospheric pressure. Immunocytochemical analysis with iso[4]LGE_2_–protein adduct antibody was carried out for control cells (**B**) and for astrocytes (**C**) that were subjected to 3 h of pressure at 100 mmHg and then subsequently subjected to a recovery period of 16 h.

Glaucomatous individuals often possess elevated IOP. In patients with POAG, the IOP can reach 23–25 mmHg while patients with pigmentary and other secondary glaucomas often show higher pressure. In extremely rare occasions, IOP may even reach 80 mmHg in individuals with hypertension and glaucoma. In traumatic brain injuries, especially from blasts, astrocytes could be exposed to very high pressures [16,51]. We examined whether astrocytes exposed to increased pressure develop lipid peroxidation-derived protein modifications. Isolated astrocytes were subjected to pressures ranging from 25−150 mmHg for 3 h and then allowed to settle for a period of about 16 h. Western blot analysis of cell lysates revealed that pressures of 150 mmHg and greater resulted in iso[4]LGE_2_ modifications of proteins with MWs of 50 kDa and 65 kDa (Figure 3F). Interestingly, lower pressures of 25–100 mmHg lead to a loss of iso[4]LGE_2_ modifications in proteins with an apparent MW of 35 kDa. Phalloidin staining of actin filaments shows no obvious morphological changes following pressures up to 150 mmHg (Figure 3A-E). Surprisingly, ELISA analyses designed to detect total iso[4]LGE_2_ immunoreactivity showed an increase in total iso[4]LGE_2_ immunoreactivity in pressure-treated astrocytes (Figure 4A). We reconciled the apparent discrepancy between the two data sets (Figure 3F and Figure 4A) to non-homogenous modification of proteins in this pressure range. Thus, although total modification is unaltered or may be slightly elevated as revealed by ELISA (Figure 4A), there was a lack of homogeneous modification eluding western blot detection in this pressure range when exposed for 3 h followed by a 16 h recovery period. The immunohistochemical detection of iso[4]LGE_2_ protein adduct immunoreactivity in 100 mmHg-treated astrocytes compared to controls (Figure 4B,C) was consistent with ELISA results. Immunoprecipitation of total protein lysates exposed to 25 and 100 mmHg with an antibody to iso[4]LGE_2_ protein adduct and subsequent mass spectrometry identified several potential iso[4]LGE_2_-modified proteins that are involved in various cellular processes (Table 1). All proteins identified in Table 1 were obtained as a result of two independent immunoprecipitation (IP) experiments.

**Table 1:** Identification of proteins after anti-iso[4]LGE_2_ immunoprecipitation.

Accession number*	**Description**	**Peptide matches**	**MW (Da)**
P11844	Gamma crystallin A	2	20,877
P02511	Alpha-crystallin B chain	2	20,159
P53672	Beta crystallin A2	2	22,096
P30154	Serine/threonine protein phosphatase 2A	2	66,183
P02489	Alpha-crystallin A chain	2	19,909
P01730	T-cell surface glycoprotein CD4	2	51,111
Q04759	Protein kinase C, theta type	2	81,847
P01236	Prolactin	2	25,876
P02746	Complement C1q subcomponent	2	26,441
P01137	Transforming growth factor beta-1	2	44,341
Q2L1Q8	MHC class II antigen	2	28,166
Q01094	Transcription factor E2F1	2	46,920
B1ALM2	Calcium channel, voltage-dependent	2	212,350
P11511	Cytochrome P450 19A1	2	57,883
P54687	Branched-chain-amino-acid aminotransferase, cytosolid	2	42,934
P03956	Interstitial collagenase	2	54,007
Q6Y4Q7	Tumor necrosis factor receptor superfamily member 1A	2	4,697
Q92674	Leucine-rich primary response protein 1	2	86,699
Q14830	Serine/threonine protein phosphatase with EF-hands-2	2	86,413
Q02779	Mitogen-activated protein kinase 10	2	103,605
Q9UHL4	Dipeptidyl-peptidase II precursor	2	54,309

The asterisk indicates that the column displays SWISS-PROT accession numbers of identified proteins. Astrocytes were subjected to pressure treatment for 4 h at 100 mmHg. Proteins from these astrocytes were identified by mass spectrometry. Proteins that were identified in at least two independent experiments have been presented.

### Increased iso[4]LGE_2_ protein modification in astrocytes exposed to high pressures for longer durations

**Figure 5:**
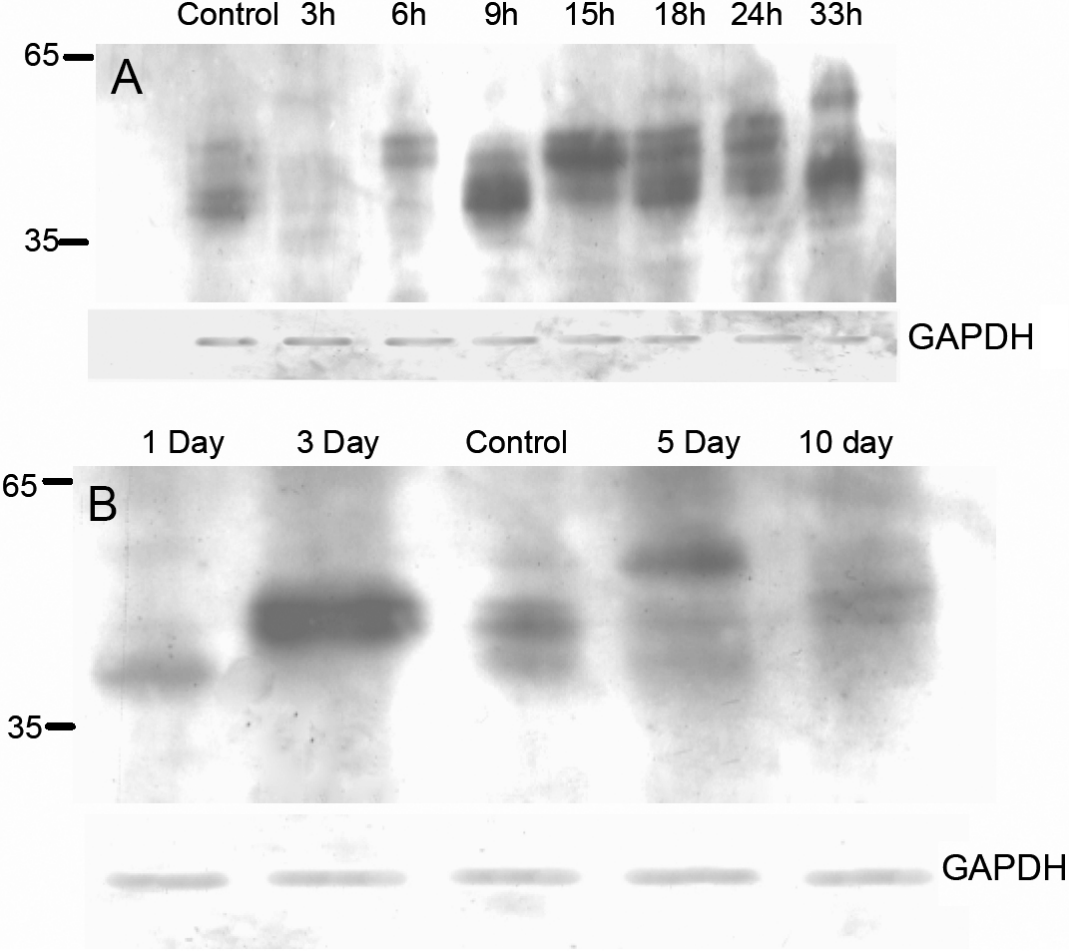
Western blot analyses of protein extracts from astrocytes. A representative figure from three repeating experiments is shown. **A**: Analysis is shown of protein extracts from rat brain cortex astrocytes that were subjected to different durations of pressure. Control cells were allowed to remain in the incubator at 37 °C without being exposed to any increase in pressure. Other astrocytes were subjected to an increase in pressure. Cells were subjected to fixed pressure and then analyzed for iso[4]LGE_2_–protein modification. About 3,000 isolated rat brain cortex astrocytes were subjected to a pressure of 100 mmHg for different time periods (ranging from 3–33 h). Following pressure treatment, the culture medium was immediately replaced with fresh medium, and the cells were incubated at 37 °C for 16 h at atmospheric pressure. **B**: Western blot analysis is shown of protein extracts from astrocytes that were subjected to different post pressure recovery periods for iso[4]LGE_2_ modification. About 3,000 isolated rat brain cortex astrocytes were subjected to a pressure of 100 mmHg for a period of 3 h and were then allowed to recover at 37 °C. Following pressure treatment, the culture medium was immediately replaced with fresh medium, and the cells were incubated at 37 °C for varying periods (ranging from 1–10 days) at atmospheric pressure. Western blot analysis was performed using rabbit polyclonal antibody to iso[4]LGE_2_–protein adduct after fractionation of total cell lysates (25 μg protein lysate was loaded in each lane) on 4%–20% SDS–PAGE and transfer to a PVDF membrane. In A and B, bottom panels were probed with anti-GAPDH as indicated.

Patients with glaucoma often suffer extended periods of high intraocular pressure. To determine if the increase in iso[4]LGE_2_ modifications of astrocyte proteins correlated directly with duration of pressure exposure, astrocytes were subjected to extended periods of hydrostatic pressure. Application of 100 mmHg on astrocytes for a period of 3 h−33 h showed a significant increase in iso[4]LGE_2_ immunoreactivity (Figure 5A). The cells were allowed to incubate at 37 °C overnight for about 16 h after pressure treatment for these experiments. 

### Effect of post pressure recovery period on astrocytes and prophylaxis against protein modification by endogenous iso[4]LGE_2_

**Figure 6:**
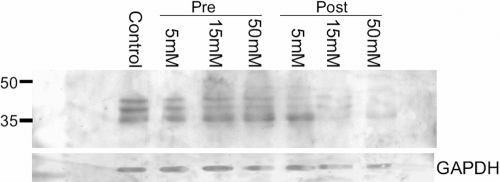
Western blot analyses for determining iso[4]LGE_2_-modification of proteins in extracts from astrocytes treated with pyridoxamine. About 3,000 isolated rat brain cortex astrocytes were treated with freshly prepared 5–50 mM pyridoxamine (except for the control sample) in water as indicated. Astrocytes were subjected to treatment with pyridoxamine before or after pressure treatment (100 mmHg for 4 h followed by a recovery period of 16 h at 37 °C and at atmospheric pressure). Western blot analysis was performed with iso[4]LGE_2_–protein adduct antibody after the transfer of 25 µg of total protein extracts onto a PVDF membrane. Bottom panel shows probing with anti-GAPDH antibody.

Glaucomatous as well as normal eyes often suffer from bouts of diurnal fluctuation in pressure, and often the pressure drops to a lower boundary spontaneously. To determine the changes in iso[4]LGE_2_ protein adduct immunoreactivity that occur during these periodic changes in pressure, we examined iso[4]LGE_2_ protein modifications in astrocytes that were subjected to a pressure of 100 mmHg for 3 h and allowed to recover for various periods (Figure 5B). Control cells were incubated for similar periods but without any pressure treatment. A representative control where cells were incubated for five days is shown (Figure 5B). In cytochemical analysis, the pressure-treated astrocytes showed elevated levels of iso[4]LGE_2_ protein adduct immunoreactivity compared to controls (Figure 4B,C). Western blot analysis showed the presence of iso[4]LGE_2_ protein adduct immunoreactivity for relatively higher molecular weight proteins even 10 days after initial pressure treatment compared to controls (Figure 5B). This data indicates that the apparent generation of iso[4]LGE_2_ protein modifications occurs and continues to increase for many days after initial pressure treatment. Prior incubation with pyridoxamine before being subjected to pressure did not show a significant effect on iso[4]LGE_2_ immunoreactivity. Post pressure treatment with pyridoxamine did decrease the formation of iso[4]LGE_2_-modified proteins (Figure 6).

## Discussion

In the present study, we subjected astrocytes to a pressure of 100 mmHg for a period of 3 h for several reasons. This pressure is easy to manage in a pressure chamber, and this pressure and the duration did not lead to any discernable changes in the medium pH or its oxygen levels during incubation [52]. A similar device have been used for hydrostatic pressure experiments by several investigators for astrocytes [44,53,54] as well as for RGC5 cell lines [55,56].

Astrocytes are generators of arachidonic and docosahexaenoic acids [41] in the brain and the optic nerve [24] and have been known to produce induced nitric oxide synthase (iNOS) within 12 h of being subjected to hydrostatic pressure [39]. Nitric oxide can also combine with superoxide to produce peroxynitrite, which can cause a large number of changes to proteins and lipids in vivo [57]. We now find that pressure-treated cells showed greater amounts of iso[4]LGE_2_ protein modifications than control cells as revealed by fluorescence microscopy (Figure 4B,C). The iso[4]LGE_2_ modification of proteins in response to pressure treatment is preventable by pharmacological intervention (Figure 6) or by long-term pressure withdrawal. However, short-term withdrawal is not very effective (Figure 5B). Whether or not such prophylaxis can be accomplished in vivo needs further investigation. Elevated levels of oxidation products of arachidonic acid such as iso[4]LGE_2_ were found in glaucomatous TM when compared to control TM [30,31] and in the optic nerve head astrocytes (Figure 2A). Previously, another lipid oxidation product, 4-hydroxynonenal, was shown to induce increased expression of antioxidant enzymes in a dose-dependent manner in optic nerve head astrocytes [58]. In vitro, an incremental increase in iso[4]LGE_2_ was observed in response to 25–40 mmHg of pressure and again beyond 100 mmHg of pressure (Figure 4A). This seems likely to be of significance for disease pathology. The cellular morphology as revealed by rhodamine-phalloidin staining appears not to undergo significant changes (Figure 3A-E). The phalloidin staining undergoes a change in intensity between 50–100 mmHg of pressure (Figure 3A-D). Western blot analysis suggests a lack of specific iso[4]LGE_2_ protein adduct immunoreactivity, but total iso[4]LGE_2_ protein adduct immunoreactivity between these pressures remains unchanged as revealed by ELISA analysis (Figure 3F and Figure 4A). The immunohistochemical observation of elevated iso[4]LGE_2_ protein adduct immunoreactivity in cells subjected to 100 mmHg of pressure was consistent with enlarged astrocytes upon being subjected to elevated pressure. Astrocytes subjected to external stimuli such as elevated pressure or hypoxia become reactive. One feature of reactive astrocytes is cell size enlargement [59,60]. An enlarged cell will have an expansion of the cell membrane thus exposing the cell membrane lipids to the oxidative environment commensurate with more iso[4]LGE_2_ protein adduct immunoreactivity at the cell surface. The cells survive for a prolonged period after pressure withdrawal (Figure 5B), i.e., a sufficiently long period of over 30 h. In our experiments, the total packed volume of cells was less than 0.5 ml, and the incubation volume was about 2 l (at least 4,000 fold more compared to cell volume). However, with extended time duration in addition to pressure, hypoxia cannot be ruled out. We determined the medium pH and oxygen content for up to 9 h of incubation. No significant changes were observed. Western blot analysis provided evidence that pressure-treated astrocytes do increase production of the specific lipid peroxidation product, iso[4]LGE_2_. Western blot analysis revealed that subjecting these cells to 100 mmHg of pressure for 3 h leads to a small decrease in iso[4]LGE_2_ after a 16 h recovery period when compared to untreated controls (Figure 5A). However, longer exposures to pressure leads to increased iso[4]LGE_2_ immunoreactivity (Figure 5A), and the recovery period also has a pronounced effect. Prolonged culture of astrocytes results in a loss of baseline iso[4]LGE_2_ immunoreactivity (data not shown). However, this loss is much less pronounced in astrocytes subjected to pressure treatment (Figure 5B).

Analysis using mass spectrometry of proteins derived from pressure-treated astrocytes revealed major potential protein targets of oxidative modification in astrocytes (Table 1). Analysis of these proteins may further elucidate mechanisms of pressure-induced damage and consequent dysfunction in astrocytes.

Two observations are especially noteworthy with respect to potential therapeutic interventions. Generation of iso[4]LGE_2_ protein modifications is a slow process that occurs after pressure has been applied and during the subsequent recovery period, and iso[4]LGE_2_ can be efficiently trapped by pyridoxamine (vitamin B6), which acts as a sacrificial nucleophile [42,61]. Thus, the application of pressure in the absence of pyridoxamine and the subsequent treatment with pyridoxamine during the recovery period dramatically reduced protein modification, presumably because pyridoxamine competes effectively with proteins for binding to isolevuglandins (isoLGs) as shown in Figure 1 [62-64].

Paradoxically, we found that the presence of pyridoxamine during the application of pressure did not reduce protein modification by the highly reactive γ-ketoaldehyde iso[4]LGE_2_, which occurred during the subsequent recovery period. This behavior is understandable if pyridoxamine is consumed by short-lived reactive oxygen species (ROS) and a torrent of electrophilic lipid oxidation products escaped from the lipid bilayer – where they are generated by oxidative cleavage of polyunsaturated acyl chains in membrane phospholipids – into the aqueous phase where pyridoxamine resides [64].

**Figure 7:**
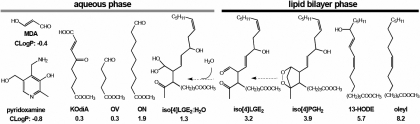
Schematic representation of phospholipids with different ClogP values, membrane-sequestered IsoPG endoperoxides, and the hydrate of iso[4]LGE_2_. Previous studies of phospholipid conformations in membranes showed that *sn*-2 acyl groups whose methyl esters have ClogP of less than 2 protrude into the aqueous phase whereas with ClogP greater than 5, they are buried in the lipid bilayer. IsoPG endoperoxides that are generated have been schematically shown (iso[4]PGH_2_ and iso[4]LGE_2_). They remain sequestered in membranes where they slowly rearrange to isoLGs that become hydrated and protrude into the aqueous phase (iso[4]LGE_2_·H_2_O) allowing them to react with pyridoxamine or proteins.

Our predictions of the preferences for lipid oxidation products to remain sequestered in the nonpolar interior of membrane lipid bilayer versus being ejected into the aqueous phase are based on previous ^1^H Nuclear magnetic Resonance (NMR) nuclear Overhauser effect studies that established the conformations of phosphatidylcholines with oxidized *sn*-2 acyl groups [65,66]. Thus, the 4-keto-2-Octene-dioic acid (KOdiA), 5-Oxovalerate (OV), and 9-Oxo-noanoate (ON) *sn*-2 acyl groups in oxidatively truncated phosphatidylcholines adopt a conformation in which these oxidized acyl groups protrude like whiskers from the lipid bilayer into the aqueous phase while an *sn*-2 13-hydroxy-9, 11-octodecadienenoate (HODE) group remains buried in the hydrophobic interior of the membrane (Figure 7). We now note that *sn*-2 acyl groups whose methyl esters have octanol/water partition coefficients, ClogP<2, protrude into the aqueous phase whereas with ClogP>5, they are buried in the lipid bilayer.

Because they are hydrophilic, some lipid oxidation products such as malondialdehyde (MDA) with ClogP=−0.36 are expected to be ejected from the lipophilic interior of the membrane, which is where they are generated, into the aqueous phase. In the aqueous phase, they can react with pyridoxamine that has ClogP=−0.81 (Figure 7) and remain there (Figure 1). A previous study showed that levels of MDA in the retina increase by an order of magnitude with a modest increase of IOP from a normal level of 14 mmHg to a modestly elevated level of 24 mmHg induced by cauterization of three episcleral veins in rats [67].

Lipid oxidation also generates prostaglandin endoperoxide isomers such as iso[4]prostaglandin endoperoxide H (PGH)_2_ that subsequently rearrange to isolevuglandins such as iso[4]LGE_2_ by a non-oxidative transformation that does not involve ROS. Even though the levels of ROS rapidly decline after the initial oxidative insult, the production of isoLGs can occur during the recovery period because their precursors, e.g., iso[4]PGH_2_, are buried in the lipophilic interior of the lipid bilayer where they very slowly transform into isoLGs (Figure 7). Thus, although prostaglandin endoperoxides rearrange rapidly (t_1/2_ ~5 min) to the γ-ketoaldehyde levuglandins in aqueous solution [68,69], rearrangement of such endoperoxides in an aprotic lipophilic environment is orders of magnitude slower [69]. The isoLGs then form hydrates [68] that are expected to protrude from the lipid bilayer into the aqueous phase where they can react with proteins or pyridoxamine (Figure 1). This scenario presents both a challenge and an opportunity to prevent pathological protein modification by isoLGs. The challenge is to withstand a protracted barrage of toxic γ-ketoaldehydes that are generated as a consequence of lipid oxidation. Their formation cannot be prevented with antioxidants applied after the generation of endoperoxide precursors because the conversion of endoperoxides into isoLGs does not involve oxidation. On the other hand, because their generation is slow, there is ample time to intercept them after the oxidative insult, and pyridoxamine is very effective in doing just that.

Subjecting astrocytes to hydrostatic pressure alters them in a way that induces oxidative injury. Oxidative stress was previously shown to be an early event in hydrostatic pressure-induced retinal ganglion cell damage [55]. The nature of the alteration remains to be determined, although disruption of membrane structures resulting in leakage [58] between cellular compartments is a potential contributor [70].

In conclusion, astrocytes isolated from the human glaucomatous optic nerve head showed higher levels of lipid-derived oxidative immunoreactivity associated with protein modifications than that of the non-glaucomatous controls. In isolated astrocytes, in vitro, the levels of modification increase with pressure. This pressure effect may account for the elevated levels of isoLG-protein modification in glaucomatous optic nerve head astrocytes compared to those in the normal nerve head. Astrocytes apparently respond to increased pressure by producing oxidation products of fatty acids such as iso[4]LGE_2_. This highly reactive lipid covalently adducts to proteins within seconds, leading to inter alia, which is the formation of protein–protein cross-links. Rhodamine-phalloidin staining revealed that astrocytes can tolerate a large amount of pressure and yet maintain their cytoskeletal structure. However, the cells have been altered, and iso[4]LGE_2 _modification of astrocyte proteins evolves with aging, favoring adducts of higher molecular weight, perhaps owing to isoLG-induced cross-linking. The ability of pyridoxamine to prevent this presumably pathological modification of proteins is a seminal discovery that seems likely to have therapeutic utility for eye and brain pathology consequent to cellular insult caused by pressure.
